# Regarding “Long-term efficacy following venous stenting for symptomatic iliofemoral venous obstruction: 5-year clinical and stent outcomes in a Southeast Asian population”

**DOI:** 10.1016/j.jvsv.2026.102474

**Published:** 2026-06-22

**Authors:** Saad Ahmad, Syed Mujtaba Hussain

**Affiliations:** Islamic International Medical College, Riphah International University, Rawalpindi, Pakistan

We read the article titled “Long-term efficacy following venous stenting for symptomatic iliofemoral venous obstruction: five-year clinical and stent outcomes in a Southeast Asian population” by Yun Sun Lim et al. This study evaluates long-term outcomes following endovenous stenting in patients with symptomatic iliofemoral venous obstruction. The authors demonstrate that dedicated venous stenting is safe and durable over a 5-year follow-up period, with high patency rates and sustained clinical improvement. The study provides valuable long-term data from a Southeast Asian population, showing improvement in pain, ulcer healing, and limb swelling. Notably, most losses of stent patency occurred early after the procedure, emphasizing the importance of procedural technique and post-procedural management.

However, we would like to argue that the need for long-term data extends beyond confirming durability alone. Most existing studies report outcomes limited to 1 or 2 years, which restricts assessment of true long-term performance.^1^ Although early results often demonstrate good patency and symptom relief, venous stents may narrow, thrombose, or fail over time. Without extended follow-up, it remains unclear whether venous stenting offers a durable solution or primarily a short-term benefit. Importantly, early patency loss may reflect anatomical mismatch rather than procedural failure. Southeast Asian patients often have smaller iliac vein diameters compared with Western populations, increasing the risk of over-stenting when Western-sized dedicated venous stents—commonly starting at 14 mm—are used. Excessive radial force and external compression in smaller veins may therefore contribute to early loss of patency, underscoring the need for long-term, population-specific registries.^2^ Moreover, Southeast Asian data are critical because pharmacological responses may differ significantly from Western cohorts.^1^ A substantial proportion of East and Southeast Asian populations carry CYP2C19 loss-of-function alleles, resulting in reduced responsiveness to clopidogrel. This may compromise standard post-stenting antiplatelet regimens and increase the risk of early thrombosis, potentially explaining early patency loss observed in some patients. Current venous stenting guidelines are largely derived from Western populations and do not account for these pharmacogenetic differences. Applying such guidelines without regional adaptation may therefore lead to suboptimal outcomes. Long-term data from Southeast Asian centers could support population-specific antiplatelet strategies and improve stent durability and safety.^3^

In conclusion, this study provides important 5-year clinical and stent outcome data for symptomatic iliofemoral venous obstruction in a Southeast Asian cohort. The findings support venous stenting as a safe and durable long-term treatment while also highlighting the importance of regional anatomical and pharmacological considerations. Further long-term studies from Southeast Asian centers are needed to refine evidence-based guidelines and improve patient outcomes.

## Declaration of generative AI and AI-assisted technologies in the writing process

During the preparation of this work, the authors used artificial intelligence tools such as deepseek, chatgpt, GROK, Gemini, Claude, and copilot solely for language refinement and to assist in identifying the most relevant supporting literature. No AI-generated content, analysis, or original ideas have been incorporated into this letter. The authors have thoroughly reviewed and verified all arguments, references, and conclusions presented herein and take full responsibility for the final content.

## Funding

None.

## Disclosures

None.
